# Acute Physiological and Emotional Responses to a Brief 24-Minute Yoga Session: Randomized Exploratory Pilot Study With Waitlist Comparison

**DOI:** 10.2196/87077

**Published:** 2026-04-13

**Authors:** Ying Zhou, Ping Chen, Yuma Morisaki, Naoko Yamada, Risa Kuwahara, Atsuro Tsutsumi, Akihiro Nomura

**Affiliations:** 1 Faculty of Health Sciences, Institute of Medical, Pharmaceutical and Health Sciences, Kanazawa University, Kanazawa, Japan; 2 Frontier Institute of Tourism Sciences, Kanazawa University, Nu 7, Kakuma-machi, Kanazawa, 9201192, Japan, 81 76-265-2259; 3 Division of Convergence Science, Kanazawa University Graduate School of Frontier Sciences, Kanazawa, Japan; 4 Department of Cardiovascular Medicine, Kanazawa University Graduate School of Medical Sciences, Kanazawa, Japan; 5 Division of Future Design, Noto Resilience and Revitalization Center, Kanazawa University, Kanazawa, Japan

**Keywords:** yoga, salivary cortisol, heart rate, electroencephalography, wellness tourism, wearables, exploratory pilot study

## Abstract

**Background:**

Wellness tourism increasingly incorporates short yoga sessions for stress management, yet evidence on their immediate physiological and emotional responses remains limited.

**Objective:**

This exploratory pilot study aimed to generate preliminary data on acute physiological and emotional responses to a single 24-minute yoga session (breathing, postures, and meditation) compared with quiet sitting in novice practitioners.

**Methods:**

In this single-center, randomized waitlist-controlled exploratory pilot study (1:1 allocation), 19 university-affiliated adults with self-reported daily stress and no prior guided yoga experience were allocated to an immediate 24-minute yoga session (n=10) or quiet sitting (n=9). The primary outcome was change in salivary cortisol from baseline to postintervention. Secondary outcomes included salivary alpha-amylase, heart rate (Apple Watch [Apple Inc], subset n=12), and real-time emotional indices (stress, calmness, and concentration) measured by a commercial single-channel electroencephalogram (EEG) device (Kansei Analyzer [Dentsu ScienceJAM], subset n=8). Data were analyzed using change scores relative to the breathing phase, with between-group comparison performed by independent *t* tests or multivariable linear regression adjusted for age, sex, and baseline values as appropriate.

**Results:**

No significant between-group differences were observed for salivary cortisol or alpha-amylase. In the yoga group, heart rate increased during postures and remained mildly elevated during meditation relative to the breathing phase, consistent with mild physical exertion. Exploratory individual EEG analyses suggested reduced stress and increased concentration during breathing and meditation phases in some participants.

**Conclusions:**

In this small exploratory pilot study, a single 24-minute yoga session did not significantly alter salivary stress biomarkers at the group level. However, it induced measurable cardiovascular activation and heterogeneous EEG-derived emotional responses. These hypothesis-generating findings demonstrate marked interindividual variability, suggesting the need for larger validation studies with improved control conditions.

## Introduction

Health tourism is a form of travel that provides nonroutine experiences aimed at restoring, maintaining, and enhancing health, typically in nature-rich environments that promote well-being [1]. The global market for health tourism is expected to reach US $411 million by 2030 [2], positioning this sector as strategically important within the broader tourism industry. Within health tourism, wellness tourism has emerged as a major subcategory because of its focus on scientifically grounded activities and programs that support physical and mental well-being [3]. Wellness tourism integrates diverse elements, including health care services, treatment and recovery programs, relaxation techniques, dietary therapy, and physical exercise during travel experiences [1,4].

As global awareness of mental and physical self-care rises, wellness tourism is increasingly sought by travelers desiring physical renewal, psychological balance, and personal transformation [5-7]. Yoga retreats, a key modality within wellness tourism, offer structured programs that blend movement, breathwork, and meditation in calming natural settings. These programs promise not only relaxation and stress relief but also opportunities for deep emotional engagement and transformation [8].

As a key component of wellness tourism, yoga is increasingly recognized for its ability to meet the needs of travelers seeking to improve physical and mental health and achieve spiritual fulfillment [9]. Frequently incorporated into wellness tourism programs, yoga offers a combination of physical and mental health benefits through practices such as *Pranayama* (breathing techniques), *Asanas* (physical postures), and *Dhyana* (meditation) [10]. Given that chronic stress is a major contributor to numerous physical and mental health issues, including cardiovascular diseases, anxiety disorders, and depression [11], these yoga practices are expected to reduce chronic stress, improve mental health, and promote overall well-being [10]. Thus, integrating yoga into wellness tourism programs could have the potential to enhance their effectiveness in promoting long-term health and wellness.

Despite the increasing popularity of yoga in wellness tourism, further scientific evidence is required to validate its stress-reduction effects, particularly its immediate short-term effects, in various contexts. Previous studies have compared the effects of yoga on stress reduction, mental health, and physiological responses, frequently comparing yoga practitioners with control groups using subjective assessments and objective biomarkers. Subjective assessments, such as the Perceived Stress Scale (PSS) and self-reported emotional state surveys, consistently emphasize the effectiveness of yoga in decreasing perceived stress and improving well-being [12]. However, participants’ expectations or response biases can influence subjective evaluations, highlighting the need to complement these assessments with objective physiological indicators to increase the reliability of analysis. Objective studies provide evidence supporting the physiological effects of yoga. For example, yoga interventions have been associated with decreased cortisol levels, which are a primary stress biomarker [13]; improvements in heart rate variability, indicating improved balance of the autonomic nervous system [14]; and decreases in salivary alpha-amylase (sAA) levels, reflecting decreased activities in the sympathetic nervous system and attenuated stress responses [15]. However, many of these studies used a nonrandomized design and focused on the long-term effects of sustained yoga practice, leaving its short-term effects under controlled conditions largely unexplored.

To address this gap and strengthen the evidence base for yoga programming in wellness tourism, we conducted a randomized waitlist-controlled design to investigate the acute physiological and emotional effects of a brief yoga session. Additionally, we used a novel approach by integrating traditional biomarkers with real-time emotional analysis using the Kansei Analyzer (Dentsu ScienceJAM) [16,17], an ambulatory electroencephalogram (EEG) device designed to objectively measure emotional variability. By combining traditional biomarkers with this advanced emotional analysis device, this study aimed to provide new insights into the acute stress-reduction effects of yoga. This approach may reinforce the importance of incorporating yoga into wellness tourism programs and offer practical, evidence-based information for practitioners and policymakers in the field. The specific objectives were to test the hypothesis that a brief yoga session would reduce salivary cortisol (primary) and improve secondary outcomes (sAA, heart rate [HR], emotional states) compared to waitlist control, while exploring individual variability.

## Methods

### Study Design

**Figure 1.**
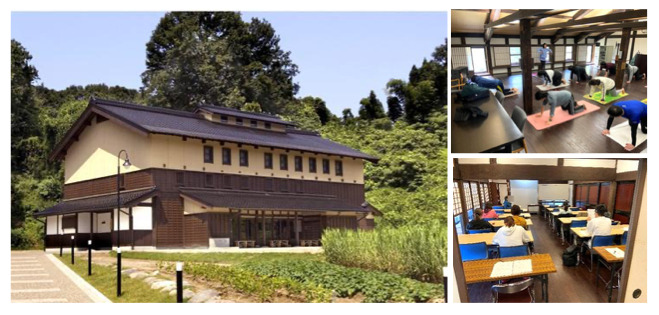
The study site. Left: Exterior view of the facility in a quiet, natural setting. Top right: Second-floor activity room used for yoga sessions. Bottom right: First-floor conference room used as a resting space.

This single-center, prospective, randomized, waitlist-controlled, exploratory pilot study assessed the acute physiological effects of a brief yoga intervention (eg, breathing exercises, poses, and meditation) on stress markers. This study was conducted on a single day. Participants were randomly assigned to a yoga group, which performed the yoga program, or a waitlist control group, which rested quietly in a seated position during the intervention period. Subsequently, the participants completed a baseline questionnaire providing demographic and lifestyle data, including age, sex, occupation, living situation (alone or with others), presence of comorbidities, alcohol consumption habits, and smoking habits. The participants also completed the Japanese version of the PSS (PSS-14) to assess their perceived stress levels before the intervention. The validity and reliability of the Japanese version of the PSS-14 have been previously established [18]. The study was conducted at Kakuma no Sato Kanazawa University, a 2-story building situated in a quiet, natural environment away from road traffic, surrounded by greenery and providing a calm, retreat-like atmosphere. The first floor was allocated to the waitlist control group to ensure a calm resting environment, whereas the second floor was designated for the yoga intervention group and provided adequate space for movement and focus (Figure 1).

### Ethical Considerations

All procedures adhered to the Ethics Guidelines for Medical and Biological Research Involving Human Subjects in Japan [19]. The study was approved by the Medical Ethics Committee of Kanazawa University (2023‐250, Receipt Number: 114441). Before randomization, an explanatory session was held to provide details on the procedures and obtain written informed consent. The trial design was parallel group with a 1:1 allocation ratio. No important changes to methods occurred after trial commencement. Participant privacy and confidentiality were strictly protected. No personally identifiable information was collected beyond what was necessary, and all data were anonymized during analysis and reporting. Participants received a 1000 Japanese yen (US $0.0063) shopping card as compensation for their time and participation.

### Study Participants

We recruited the staff, students, and their families at Kanazawa University as participants via the internal communication system of the university. The inclusion criteria were as follows—participants (1) aged 18 years or older at the time of consent (no sex restriction), (2) who self-reported subjective daily mental and physical stress, (3) with interest in participating in stress-management yoga programs, and (4) with no previous experience in professionally instructed yoga or meditation programs. The exclusion criteria were as follows: participants (1) who could not complete salivary tests; (2) who were unable or unwilling to wear an Apple Watch (Apple Inc) (provided by researchers); (3) who could not undergo the Kansei Analyzer evaluation, as determined by the principal investigator or coinvestigators; and (4) who did not provide written informed consent. Participants received a 1000 Japanese yen (US $0.0063) shopping card as compensation for their time and participation. As this was a pilot study, no formal sample size calculation was performed; recruitment aimed for 20 participants to assess feasibility and generate preliminary data for future trials.

### Yoga and Waitlist Control Groups

The 24-minute duration was deliberately chosen to mimic brief yoga sessions commonly offered in wellness tourism settings (eg, hotel morning classes, airport yoga rooms, or short retreat modules), where time constraints are typical, rather than to replicate full-length retreat sessions. The sequence (breathing → postures → meditation) and phase duration were designed to reflect a balanced, beginner-friendly practice while allowing examination of phase-specific responses.

**Figure 2.**
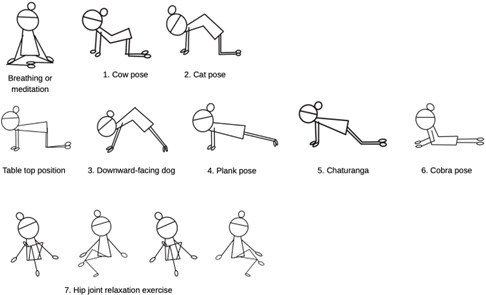
Illustration of the yoga pose flow.

Participants in the yoga group engaged in a 24-minute session led by a certified instructor (RK; Figure 2). This session commenced with approximately 5 minutes of *Pranayama* (breathing exercises), which is characterized by focused nasal breathing comprised of a controlled 4-count inhalation and 4-count exhalation. This exercise yields approximately 3‐4 breaths per minute and emphasizes diaphragmatic movement (abdominal expansion during inhalation and contraction during exhalation). After the breathing exercises, the participants performed approximately 15 minutes of *Asanas* (yoga poses). This sequence included cow pose (Bitilasana), cat pose (Marjaryasana), downward-facing dog (Adho Mukha Svanasana), plank pose (Phalakasana), a knee-down variation of Chaturanga Dandasana, cobra pose (Bhujangasana), and a concluding hip joint relaxation exercise that involves gentle leg swaying (Figure 2). The asana sequence was designed to be light-to-moderate intensity, typical for beginner Hatha yoga. The yoga session concluded with approximately 4 minutes of *Dhyana* (meditation) in a seated Siddhasana (Accomplished Pose), guiding participants to focus on natural breathing while maintaining an upright spinal posture. After completing the yoga session, saliva tests were then conducted for the yoga group (as an initial evaluation period).

Concurrently, participants in the waitlist control group engaged in a 24-minute period of quiet, seated rest. During this time, activities such as reading or using mobile phones were prohibited to maintain a restful state comparable to the nonphysical aspects of the start and end of the yoga intervention. After finishing this rest period and the subsequent data collection (as the initial evaluation period), the waitlist control group participated in an identical 24-minute yoga session (including breathing, poses, and meditation) led by the same certified instructor (LK) who guided the yoga group. After completing the session, saliva tests were also conducted (as the second evaluation period).

### Outcomes

**Figure 3.**
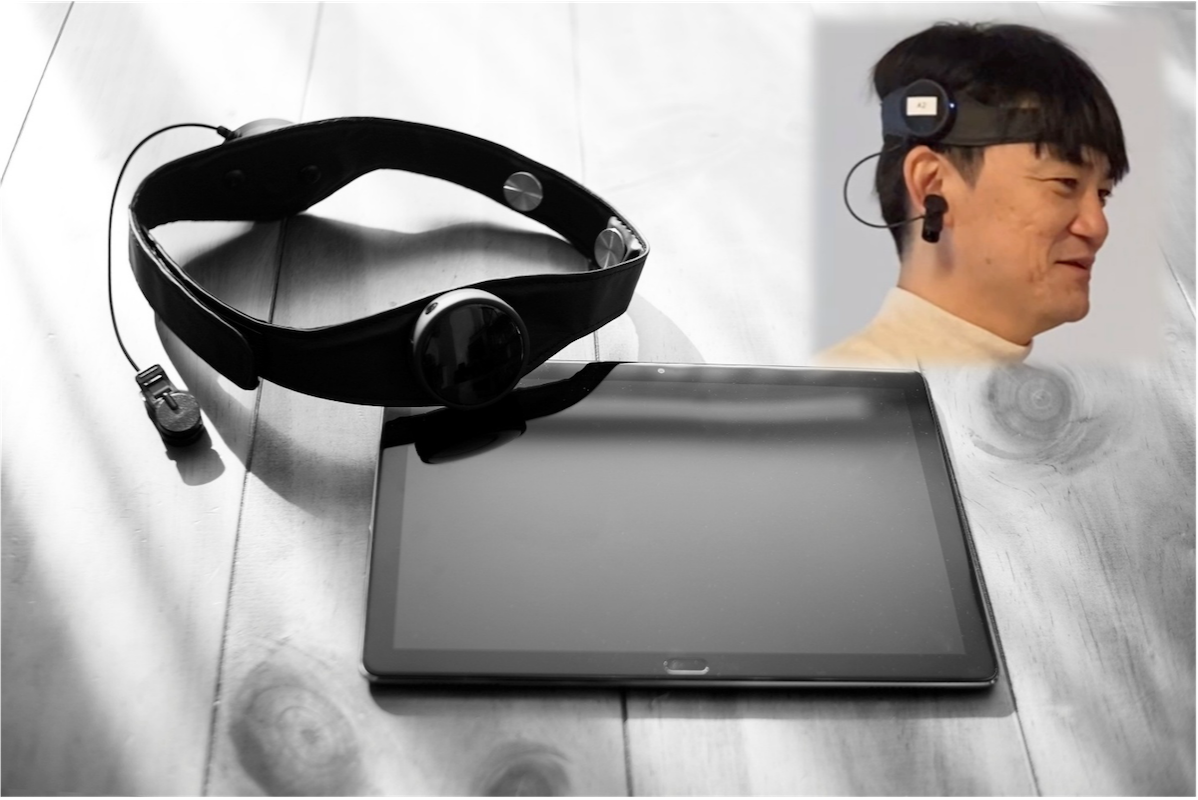
Kansei Analyzer. Left: Kansei analyzer headset and tablet for monitoring emotional status. Top right: example of how the device is worn. The individual depicted provided informed consent for publication of the image.

The study evaluated primary and secondary outcomes, which were measured at different time points relative to the intervention period (before, during, and after). The primary outcome was the change in salivary cortisol levels, a stress biomarker related to hypothalamic–pituitary–adrenal axis activity, during the initial evaluation period. The secondary outcomes included changes in salivary cortisol levels during the second evaluation period and sAA levels, a marker of sympathetic nervous system activity, during the initial and second evaluation periods for the waitlist control group. We also assessed within-group changes of these salivary biomarkers during the initial and second evaluation periods. Furthermore, the study monitored real-time physiological and emotional changes during the sessions as secondary outcomes: HR was tracked using Apple Watch devices, while emotional status (ie, stress, calmness, and concentration) was assessed using the Kansei Analyzer (Figure 3). While salivary measures (cortisol and sAA) were obtained from all participants, data collection using HR and the Kansei Analyzer was limited to subsets due to equipment availability. No changes to trial outcomes were made after commencement.

### Randomization and Blinding

Randomization was performed using a manual allocation method. Group assignments (yoga or waitlist control, 1:1 allocation) and device assignments (Apple Watch or Kansei Analyzer, due to limited availability) were designated using codes (eg, C1, C2, C3 for waitlist control; Y1, Y2, Y3 for yoga group; with additional codes indicating device allocation) written on cards and placed into opaque sealed envelopes by YZ (study coordinator). Participants were enrolled by AN (researcher responsible for enrollment), and each participant randomly drew an envelope to determine their group and device assignment. Assignments were revealed immediately upon drawing and recorded by YZ. Due to the behavioral nature of the intervention, participants and instructors were not blinded. However, laboratory technicians analyzing saliva samples and data analysts for saliva, HR, and Kansei Analyzer outcomes were blinded to group assignment. Blinding of objective outcomes (saliva, HR, and Kansei Analyzer) minimized bias. The trial was not prospectively registered because it was a pilot study, which is common for exploratory studies.

### Saliva Analysis

We collected saliva samples from all participants before and after the initial intervention/rest period. An additional post-yoga saliva sample was then collected from the waitlist control group after the completion of the subsequent yoga session. Salivary cortisol and sAA concentration levels were measured using kits from Salimetrics (State College). Salimetrics via a Japanese intermediary performed the measurements, and samples were transported frozen. Final concentrations were based on duplicate measurements per sample. Saliva collection followed the procedures outlined in the *Saliva Collection Handbook* [20]. The participants were instructed to refrain from dental treatment within 24 hours; report consumption of alcohol, caffeine, nicotine, or medication within 12 hours; avoid eating or drinking for 60 minutes, avoid brushing their teeth for 45 minutes prior to collection; and disclose any oral blood contamination. These instructions were provided via e-mail and reiterated at briefing prior to the experiment. Samples were collected using the passive drool method with collection tubes from Salimetrics (State College). To minimize the influence of diurnal variation, all collections were conducted between 2:00 PM and 3:30 PM. We placed the collected samples in an ice-packed container (4 °C for up to 2 h) and transported them to a freezer with a temperature of −20 °C at Kanazawa University, which adheres to storage guidelines for delayed freezing [20].

### Heart Rate Analysis

HR was continuously monitored using Apple Watch devices worn on the wrists of the participants. Falter et al [21] previously validated the accuracy of Apple Watches for HR monitoring. Data were exported for analysis of changes in HR during the rest and yoga phases. As 12 Apple Watches were available, the devices were randomly assigned to 6 participants each in the yoga and waitlist control groups during the initial intervention or rest period. In the second period, the waitlist control group practiced yoga using the same 6 devices they had previously used during the first period as controls. Thus, HR data during yoga practice were obtained from a total of 12 participants (n=6 from the initial yoga group and n=6 from the waitlist control group during their yoga session), and HR data during quiet rest were obtained from 6 participants from the waitlist control group during the initial period.

### Kansei Analyzer (EEG-Based Emotional State Analysis)

The study used the Kansei Analyzer to assess real-time emotional status [16]. This lightweight device uses a single electrode placed on the left forehead (Fp1, according to the International 10‐20 system) and a reference electrode attached to the left earlobe (Figure 3). Brainwave data are recorded at 128 Hz and analyzed in real-time to estimate levels of liking, interest, concentration, calmness, and stress on a calibrated scale of 0‐100 for each patient [16,17]. This study focused on stress, calmness, and concentration.

Four units of the Kansei Analyzer were used in the study. During the initial evaluation period, 1 device was assigned to a randomly selected participant from the yoga group and 3 devices were assigned to randomly selected participants from the waitlist control group. During the second evaluation period (when the waitlist control group performed yoga), the 3 participants in the waitlist control group who previously wore the device during the rest period continued to wear them. Additionally, the device previously used by the participant from the yoga group was reassigned to a fourth randomly selected participant from the waitlist control group. Consequently, data from the Kansei Analyzer during yoga were obtained from 5 participants (n=1 from the yoga group during the initial evaluation period and n=4 from the waitlist control group during their second evaluation period). Moreover, data during the rest period were obtained from 3 participants from the waitlist control group during the initial evaluation period. Explicit written informed consent for publication of the image in Figure 3 was obtained from the individual depicted.

### Statistical Analysis

To address potential concerns about repeated measures being treated as independent observations, change scores relative to the breathing phase (Δ breathing-to-posture and Δ breathing-to-meditation) were calculated for heart rate and Kansei Analyzer outcomes. This ensured that each participant contributed only one value per phase interval in the primary between-group comparisons.

The collected data were checked for completeness and consistency before analysis. Descriptive statistics (means and SDs for continuous variables; frequencies and proportions for categorical variables) for baseline characteristics and outcomes were calculated for both groups. To assess the primary and secondary outcomes related to salivary biomarkers (cortisol and sAA), pre–post intervention changes from baseline to the initial evaluation period were compared between the yoga and waitlist control groups using independent *t* tests. Within-group analyses were also performed for each group in the initial (both groups) and second (waitlist control group) evaluation periods using paired *t* tests. Regarding the assessment of HR changes, we performed multivariable linear regression adjusted for age, sex, and baseline HR. Two change metrics were calculated: changes (a) in the breathing-to-posture (B–P) interval (HR during the posture phase minus HR during the breathing phase) and (b) in the breathing-to-meditation (B–M) interval (HR during the meditation phase minus HR during the breathing phase). The regression model predicted changes in HR (B–P and B–M intervals) using group assignment (combined yoga group vs the waitlist control group) as the primary predictor. Additionally, we performed within-group comparisons of HR across the breathing, posture, and meditation phases using paired *t* test for both groups. For emotional responses measured using the Kansei Analyzer, changes in scores for stress, calmness, and concentration were compared between the combined yoga group (n=5) and the waitlist control group (n=3) using the same B–P and B–M intervals used for HR. Within-group comparisons of stress, calmness, and concentration across the breathing, pose, and meditation phases were also performed using the paired *t* test. Exploratory individual case analyses were performed using paired *t* test to compare emotional indicators (stress, concentration, and calmness) counted per minute between the yoga and sitting conditions for each corresponding interval (grouped by breathing [1‐5 min], posture [6‐20 min], and meditation [21‐24 min]) for each individual who participated in both conditions. Statistical analyses were performed using R (version 4.4.2; R Foundation for Statistical Computing) and SPSS Statistics (IBM Corp). Additional analyses (eg, individual cases) were exploratory and distinguished as such. Given the exploratory nature of this pilot study and the small sample size, *P* values less than .05 were considered noteworthy rather than statistically significant. The study is reported in accordance with the CONSORT checklist (Checklist 1).

## Results

### Participant Flow and Recruitment

**Figure 4.**
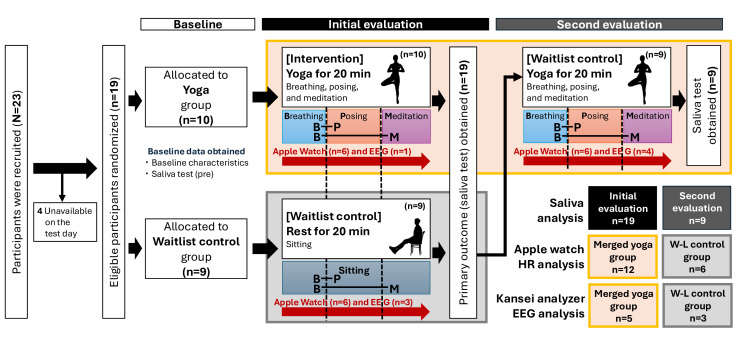
Flow diagram of the study. EEG: electroencephalogram; HR: heart rate.

Recruitment occurred from March 1 to March 22, 2024. Of the 23 applicants, 4 were unable to participate due to scheduling conflicts, and 19 were eligible and randomized (n=10 to yoga and n=9 to waitlist control). All randomized participants received their allocated intervention and were analyzed for the primary outcome (no losses or exclusions after randomization; see Figure 4 for flow). The trial ended as planned after data collection.

### Baseline Characteristics

The baseline characteristics of the participants are presented in Table 1. The mean ages of the participants in the yoga and waitlist control groups were 29.0 (SD 10.4) and 40.1 (SD 16.6) years, respectively. In the yoga group, 50% (5/10) of the participants were female compared with 67% (6/9) in the waitlist control group. No participants reported comorbidities, such as hypertension, diabetes, or dyslipidemia. Moreover, baseline PSS-14 scores, salivary cortisol levels, and sAA levels were well-balanced between the groups.

**Table 1.** Baseline characteristics of the participants.

Characteristics	Total (n=19)	Yoga group (n=10)	Waitlist control group (n=9)
Age (y), mean (SD)	34.3 (14.4)	29.0 (10.4)	40.1 (16.6)
Female, n (%)	11 (58)	5 (50)	6 (67)
Social factor, n (%)			
Living together	11 (58)	5 (50)	6 (67)
Living alone	8 (42)	5 (50)	3 (33)
Comorbidities, n (%)			
Hypertension	0 (0)	0 (0)	0 (0)
Diabetes mellitus	0 (0)	0 (0)	0 (0)
Dyslipidemia	0 (0)	0 (0)	0 (0)
Alcohol, n (%)			
None	8 (42)	4 (40)	4 (44)
Occasional	10 (53)	6 (60)	4 (44)
Daily	1 (5)	0 (0)	1 (11)
Smoking, n (%)			
Never	18 (95)	10 (100)	8 (89)
Former	1 (5)	0 (0)	1 (11)
Current	0 (0)	0 (0)	0 (0)
PSS^a^, mean (SD)	23.6 (6.9)	22.9 (6.8)	24.4 (7.3)
High-PSS group (29-56), n (%)	6 (32)	3 (30)	3 (33)
Low-PSS group (0‐28), n (%)	13 (68)	7 (70)	6 (67)

a PSS: Perceived Stress Scale.

### Saliva Analysis

For the primary outcome, changes in pre–post salivary cortisol levels from baseline to the initial evaluation period were not significantly different between both groups (mean difference: 0.033; 95% CI −0.057 to 0.12; *P*=.48). For the secondary outcome, changes in pre–post sAA levels also did not exhibit significant differences between the groups (mean difference: 2.9; 95% CI −63 to 69; *P*=.93; Table 2). Additionally, no significant within-group differences in salivary cortisol and sAA levels were observed from baseline to the initial or second evaluation period between the groups (Table 2). All 19 participants were included in these analyses (intention-to-treat).

**Table 2.** Changes in salivary cortisol and alpha-amylase levels from baseline to each evaluation period between and within groups.

	Baseline vs initial evaluation	Baseline vs second evaluation
Between-group difference		
Salivary cortisol, mean change (95% CI); *P* value	0.033 (−0.057 to 0.12); *P*=.48	—^a^
Salivary α-amylase, mean change (95% CI); *P* value	2.9 (−63 to 69); *P*=.93	—
Within-group change		
Yoga group (n=10)		
Salivary cortisol, mean change (SE); *P* value	0.029 (0.03); *P*=.37	—
Salivary α-amylase, mean change (SE); *P* value	−36 (24); *P*=.16	—
Waitlist control group (n=9)		
Salivary cortisol, mean change (SE); *P* value	−0.003 (0.025); *P*=.91	0.055 (0.061); *P*=.39
Salivary α-amylase, mean change (SE); *P* value	−32 (16); *P*=.09	−18 (39); *P*=.65

a Not available.

### Heart Rate Responses

To evaluate the immediate physiological effects of the yoga intervention, we analyzed changes in HR. For between-group comparisons, the combined yoga group exhibited an increase in HR in the B–P interval (adjusted difference: 15.5; 95% CI 10.7-20.2; *P*<.001) and B–M interval (adjusted difference: 5.1; 95% CI 0.87-9.2; *P*=.02; Table 3) compared with that in the waitlist control group. Within-group comparisons revealed increased HR in the combined yoga group during the B–P (*P*<.001) and B–M (*P*=.007) intervals, whereas the waitlist control group showed no significant changes in either interval (Table 3).

**Table 3.** Changes in heart rate and emotional state during yoga session or resting control between and within groups.

	Breathing vs Posture (B–P)	Breathing vs Meditation (B–M)
Between-group difference		
HR^a^ responses (n=18)		
HR change estimate, β (95% CI); *P* value	15.5 (10.7 to 20.2; *P*<.001)	5.1 (0.87 to 9.2); *P*=.02
Emotional state analysis (n=8)		
Stress, mean change (95% CI); *P* value	6.5 (−11.3 to 24.4); *P*=.38	−6.8 (−24.0 to 10.4); *P*=.31
Concentration, mean change (95% CI); *P* value	−10.7 (−21.6 to 0.28); *P*=.054	−5.5 (−15.8 to 4.8); *P*=.22
Calmness, mean change (95% CI); *P* value	1.5 (−1.7 to 4.6); *P*=.28	2.0 (−2.4 to 6.4); *P*=.30
Within-group change		
Combined yoga group		
HR (n=12), mean change (SE); *P* value	13.9 (1.2); *P*<.001	3.5 (1.1); *P*=.007
Stress (n=5), mean change (SE); *P* value	5.2 (4.1); *P*=.28	−6.8 (2.7); *P*=.07
Concentration (n=5), mean change (SE); *P* value	−11 (4.0); *P*=.05	10.8 (2.7); *P*=.02
Calmness (n=5), mean change (SE); *P* value	−2.2 (1.2); *P*=.13	0.0 (1.6); *P*>.99
Waitlist control group		
Heart rate (n=6), mean change (SE); *P* value	−1.5 (1.7); *P*=.39	−1.4 (1.5); *P*=.39
Stress (n=3), mean change (SE); *P* value	−1.3 (5.2); *P*=.82	0.0 (4.9); *P*>.99
Concentration (n=3), mean change (SE); *P* value	−0.3 (1.8); *P*=.87	16.3 (3.0); *P*=.03
Calmness (n=3), mean change (SE); *P* value	−3.7 (0.3); *P*=.008	−2.0 (0.6); *P*=.07

a HR: heart rate.

### Real-Time Emotional State Analysis (Kansei Analyzer)

For emotional state analysis, we observed no significant between-group differences for stress, concentration, or calmness during B–P or B–M intervals. For within-group analysis, changes in concentration increased during the B–M interval for the combined yoga (*P*=.02) and control (*P*=.03) groups. Moreover, stress and calmness exhibited no significant changes in either group, except for a decrease in calmness during the B–P interval in the waitlist control group (*P*=.008; Table 3).

**Figure 5.**
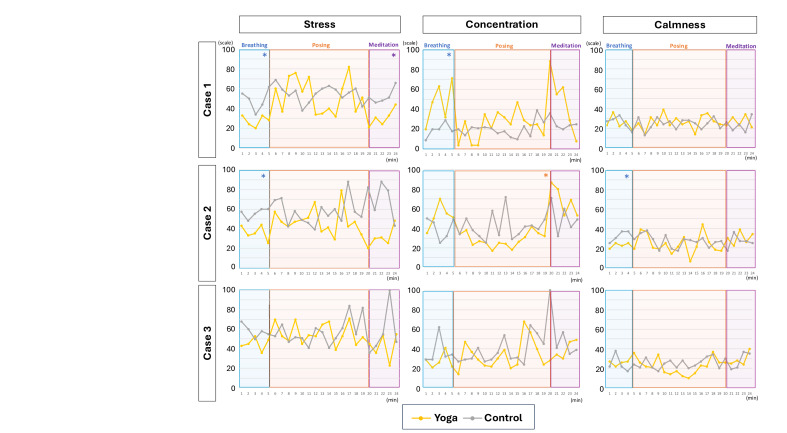
Changes in emotional status during yoga and sitting sessions in participants receiving interventions. *P<.05.

We further performed exploratory analyses of continuous emotional variations in the 3 individuals from the waitlist control group who were equipped with the Kansei Analyzer and participated in the sitting and yoga sessions (Figure 5). For individual 1, participating in yoga was associated with lower stress levels during the breathing (*P*=.007) and meditation (*P*=.002) phases than in the rest session. This individual also exhibited high scores in concentration during the breathing phase compared with those in the rest session (*P*=.04). For individual 2, stress levels decreased during the breathing phase relative to the rest phase (*P*=.007); however, a decrease in calmness was also observed during the same phase (*P*=.007). Additionally, a decrease in concentration was observed during the posture phase (*P*=.02) compared with the corresponding phases during the sitting position. In contrast, no significant differences were observed between the yoga and sitting sessions for individual 3 across the 3 parameters of emotional status. Individual EEG analyses suggested stress reduction in some participants. These individual EEG analyses were exploratory in nature. No harms or unintended effects were reported in either group.

## Discussion

### Principal Findings

This exploratory pilot study investigated acute physiological and emotional responses to a single 24-minute yoga session in novice practitioners using a randomized waitlist-controlled design. No significant group-level reductions were observed in salivary cortisol or alpha-amylase. Heart rate increased during the posture phase and remained mildly elevated during meditation relative to the breathing phase in the yoga condition, consistent with the mild-to-moderate physical exertion expected in beginners. Exploratory individual EEG analyses using a commercial single-channel device suggested possible reductions in stress and increases in concentration during the breathing and meditation phases in some participants, although responses were highly heterogeneous and subsample sizes were very small. The absence of detectable changes in salivary stress biomarkers aligns with previous evidence that acute, brief interventions may not consistently alter hypothalamic-pituitary-adrenal or sympathetic-adrenal-medullary axis markers immediately post intervention, particularly when measured without delayed sampling [22,23]. The timing of saliva collection (immediately after the session) may have missed potential delayed cortisol peaks. Similarly, the physical components of yoga likely contributed to the observed heart-rate elevation, which does not necessarily indicate a stress response but rather normal cardiovascular adaptation to light exercise in novices.

Real-time emotional monitoring revealed considerable interindividual variability, highlighting that breathing and meditation phases may induce favorable emotional shifts in some individuals while others show neutral or mixed responses. However, the proprietary nature of the Kansei Analyzer algorithms and the limited peer-reviewed validation of its absolute scoring preclude strong conclusions about these emotional indices.

This small exploratory pilot generates hypotheses about phase-specific and individual responses to brief yoga but provides no confirmatory evidence of stress-reduction efficacy. The findings highlight the limitations of underpowered acute-intervention studies and emphasize the need for larger trials with appropriate control conditions, validated measurement tools or biosensors, and diverse populations to support the integration of yoga into wellness tourism programs.

### Limitations and Future Directions

This exploratory pilot study has several important limitations that preclude definitive conclusions. First, the small sample size (n=19 overall; wearable subsets n=12 and n=8) and lack of formal sample-size calculation severely limited statistical power. Second, the waitlist-control design likely introduced expectancy and motivation bias, as control participants knew they would later receive yoga. Third, change scores and limited blinding of participants and the instructor may have contributed to residual bias. Fourth, salivary cortisol was measured immediately postintervention, potentially missing delayed peaks. Fifth, the Kansei Analyzer is a commercial single-channel EEG device with proprietary algorithms; its construct validity for absolute scoring of “stress,” “calmness,” and “concentration” has not been fully established in peer-reviewed literature. Sixth, the trial was not prospectively registered, and no correction for multiple comparisons was applied given the exploratory aims. This is consistent with the primary objective of pilot studies to assess feasibility and generate hypotheses. Finally, participants were university-affiliated novices and may not represent typical wellness tourists.

### Conclusions

This small exploratory pilot study found no significant group-level reductions in salivary cortisol or alpha-amylase following a single 24-minute yoga session compared with quiet sitting. Heart rate elevation during postures and meditation was consistent with mild-to-moderate physical exertion in novice practitioners. While EEG-derived emotional indices suggested potential stress reduction and increased concentration during breathing and meditation in some participants, marked interindividual variability was observed. Overall, these hypothesis-generating findings demonstrate the complexity of assessing acute interventions and emphasize the need for larger validation studies with improved control conditions.

## Supplementary material

10.2196/87077 Multimedia Appendix 1 Research protocol for yoga.

10.2196/87077 Checklist 1 CONSORT checklist.

## Abbreviations

B–M: breathing-to-meditation
B–P: breathing-to-posture
CONSORT: Consolidated Standards of Reporting Trials
EEG: electroencephalogram
HR: heart rate
PSS: Perceived Stress Scale
sAA: salivary alpha-amylase
