# Supplementary material for: Acute Physiological and Emotional Responses to a Brief 24-Minute Yoga Session: Randomized Exploratory Pilot Study With Waitlist Comparison
Source: JMIR Form Res. 2026 Apr 13;10:e87077. doi: 10.2196/87077 (PMC13075637; doi:10.2196/87077)
Supplement: Multimedia Appendix 1 [file formative-v10-e87077-s001.docx]

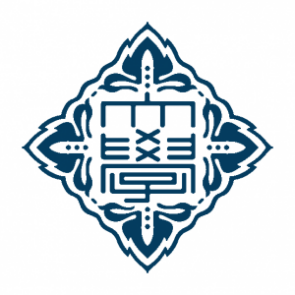


**ヨガ瞑想・呼吸法を介した**

**生体内ストレスの経時的・生理学的変化の検討**

**研究計画書**

**研究責任者**

金沢大学融合研究域融合科学系 / 先端観光科学研究所 / 附属病院循環器内科

野村 章洋

第1.1版 　2024年2月22日

目次

[１．課題名 3](#_Toc144734369)

[２．研究の概要・目的・意義 3](#_Toc144734370)

[３．研究の科学的合理性と根拠 4](#_Toc144734371)

[４．研究対象者の選定方針 4](#_Toc144734372)

[５．目標数と研究実施期間 4](#_Toc144734373)

[６．研究方法 5](#_Toc144734374)

[７．観察・検査・報告項目 6](#_Toc144734375)

[８．有害事象の評価と報告 7](#_Toc144734376)

[９．評価項目(アウトカム) 7](#_Toc144734377)

[１０．統計的事項 7](#_Toc144734378)

[１１．症例報告書の記入と報告 7](#_Toc144734379)

[１２．倫理的配慮 7](#_Toc144734380)

[１３．インフォームド・コンセントを受けるための手続きについて 8](#_Toc144734381)

[１４．研究対象者に生じる費用負担について 8](#_Toc144734382)

[１５．本研究に係る資金ならびに利益相反について 8](#_Toc144734383)

[１６．実施計画の変更について 8](#_Toc144734384)

[１７．試料・情報について 9](#_Toc144734385)

[１８．部局長への報告 9](#_Toc144734386)

[１９．研究成果の帰属と結果の公表 10](#_Toc144734387)

[２０．研究実施体制 10](#_Toc144734388)

[２１．研究業務の一部を委託する場合の当該業務内容及び委託先の監督方法 10](#_Toc144734389)

[２２．モニタリングについて 10](#_Toc144734390)

[２３．監査について 10](#_Toc144734391)

[２４．相談窓口 10](#_Toc144734392)

[２５．文献 10](#_Toc144734393)

# **１．課題名**

　ヨガ瞑想・呼吸法を介した生体内ストレスの経時的・生理学的変化の検討

# **２．研究の概要・目的・意義**

　ヘルスツーリズムとは、「自己の自由裁量時間の中で、日常生活を離れて、主に特定の地域に滞在し、医科学的な根拠に基づく健康回復・維持・増進に繋がり、かつ楽しみの要素がある非日常的・異日常的な体験を行い、必ず居住地に帰ってくる活動」と定義される。具体的には自然豊かな地域を訪れ(exercise program)、そこにある温泉を楽しみ(hydrotherapy)、体に良い料理を味わい(spa cuisine)、心身ともに癒やされる(body treatment)ことでウェルビーイングを得るものであり、疾患のある方を対象とした医療に近いメディカルツーリズムと、レクリエーション・レジャーの要素が強いウェルネスツーリズムの両方を包括する**^文献1^**。ヘルスツーリズム全体の市場規模は2030年には全世界で9兆円超(＄93.9B)に達すると推定され、今や観光業界において21世紀の重要な位置を占める旅行形態となっている。日本においてもヘルスツーリズムという言葉ができるはるか昔から「湯治」の文化にみられるように、温泉を中心とした日々の疲れや病気の苦しみを癒す場が各地で育まれてきた。しかし高度成長期を経て日本の温泉旅行は、大規模な温泉宿への団体旅行と現地での宴会・レクリエーション・レジャーが主目的の「慰安旅行」としての要素が強くなり、医科学的な根拠に基づく健康回復に繋がるという理念を有するヘルスツーリズムとは大きくかけ離れたものとなってしまった。さらに日本におけるバブル崩壊と景気低迷による企業主導の慰安旅行の縮小や廃止、個人の趣向と観光選択肢の多様化、さらには昨今の新型コロナウイルス感染症(COVID-19)のパンデミックにより、従来型の老朽化が進んだ温泉宿とそれを有する地域・地方は軒並み壊滅的な打撃を受ける結果となった。

　このような日本の現状とは裏腹に、近年世界各地ではヘルスツーリズムを明確に定義した上で、「Spa(スパ)」をその提供の場とした新しい観光産業として整備・発展させる動きが活発である。Spaとは、水資源を中心とした自然界が持つ力・時間・空間の作用と、健康哲学に基づいた人的なプロフェッショナル・スキルによるサービスの提供により、訪問者の精神と身体のバランスを回復し、外見・内面双方の健康・幸福感に寄与することで生きる活力をも生み出す場(Space)を指す。スパの中でも特に健康増進に関連する何らかの目標を達成するために行われる滞在型スパを「デスティネーション・スパ」と呼び、exercise program, hydrotherapy, spa cuisine, body treatmentの要素をバランスよく提供することで、外見的な美しさや減量だけでなく心身のウェルビーイングをバランス良く達成する究極のヘルスリゾートとして注目されている**^文献2^**。しかしながら、温泉を中心とした日本の温浴施設のほとんどはhydrotherapyとエステティックな意味合いの強いbody treatmentに終始しており、インバウンドの目玉となるような医学的なエビデンスを有したexercise programやspa cuisine、そしてプロフェッショナル・スキルを有した施術者によるbody treatmentといったヘルスツーリズムの要素をバランス良く提供する、確固たる健康哲学を有したデスティネーション・スパは日本ではごく一部の高級宿泊施設に限られ、極めて普及が進んでいない。また、ヘルスツーリズムの科学的根拠創出に関して、デスティネーション・スパを構成する各々の要素、あるいは要素全体が、どのように身体と精神に影響を及ぼし、結果的にウェルビーイングに至るのかを、バイオインフォマティクスとウェアラブルデバイス等のデジタル端末を用いて経時的・生理学的変化を計測することで統合的に検討するような研究は皆無である。

　以上より本研究では、このデスティネーション・スパプログラムの構成要素のうち「心身の癒やし(Body treatment)」に注目し、プロフェッショナルによるヨガセッション(瞑想と呼吸法)における、ストレス値の変化と環境の影響を、唾液検体、スマートバンド、感性アナライザを用いて測定することで、心身の癒やしに寄与する機序を多面的かつ定量的に解明する探索試験を行うことを目的とする。

# **３．研究の科学的合理性と根拠**

　ヘルスツーリズムの実装と普及には、明確な健康哲学に基づいた人的なプロフェッショナル・スキルによるサービスの提供が不可欠であるが、日本では必ずしもそのような状況にはなっていない現状がある。本研究を通して、プロフェッショナルによるヨガを介した心身の癒しの背景にある生物学的機序を解明することで、健康の再定義のみならず、幸せとは何か？という根源的な問いに対する新たな発見が得られうる。さらには、このような医学的に効果が証明されたヨガプログラムを、新たな観光資源として金沢の地に提供することができうる。

# **４．研究対象者の選定方針**

**（１）適格基準**

　1) 同意時年齢18歳以上

　2) 性別は問わない
　3) これまでインストラクターからの指導があるヨガあるいは瞑想等のプログラムへ参加したことがない

　4) 自己評価で日々心身ストレスを感じており、ヨガ・瞑想等のプログラムの参加を希望する者

**（２）除外基準**

　1) 本研究での唾液検査、スマートウォッチ、感性アナライザによる検査が難しいと研究責任者・分担者
　　 が判断した場合

2) 参加者のご同意が得られない場合

# **５．目標数と研究実施期間**

**（１）目標数: 全参加者数 20名 (うち金沢大学 20名)**

　予定症例数設定根拠: 唾液コルチーゾル測定法を用いたストレスに関する先行研究**^文献3,4^**を参考に、ヨガ瞑想を行った群の唾液中コルチゾールの変化量を -0.03 μg/dL、座位対照群の変化量を 0 μg/dL、それぞれの標準偏差を0.02とすると、サンプルサイズ計算にて単群あたり7名が必要である。これをふまえ、当日会場に現れない等の脱落を考慮し単群あたり10名、両群全体あわせて20名を必要全参加者数とした。

　なお、本研究は主に金沢大学の教職員ならびに学生を対象にアナウンスを行う。具体的にはアカンサスポータルあるいは学内のメール・通知・SNS等を介して参加者の募集を行う。なお、適格基準に合致し、除外基準に抵触しないのであれば、金沢大学の教職員あるいは学生の家族あるいは友人の参加も可とする。

**（２）研究実施期間**

予定研究期間： 承認日 ～ 2025年3月31日

うち症例登録期間： 承認日 ～ 2024年3月31日

研究実施期間： 3ヶ月間 (2024年3月31日まで)

研究結果解析期間： 1年間　(2025年3月31日まで)

# **６．研究方法**


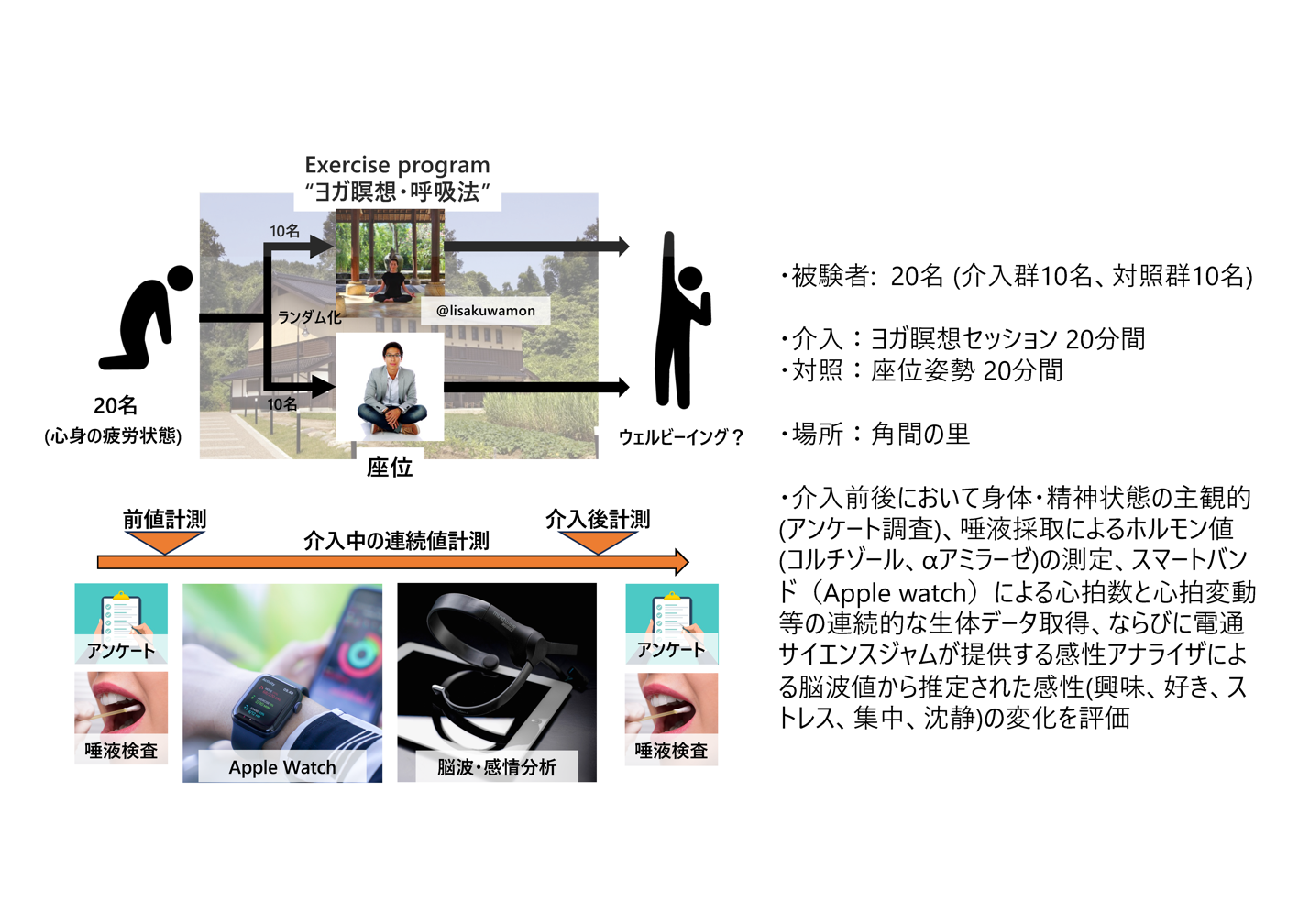
**（１）研究シェーマ**

**（２）研究のデザイン**
　単施設前向き介入研究

**（３）研究の方法**

1. 本研究は、後日指定日において、1日で行う。
2. コルチゾールの日内変動を考慮し、指定(研究)日の午後(12:00-16:00)に角間の里1階研修室に参加者20名を招集する。また唾液検査でのエラーを防ぐために、参加者は集合時間1時間前には食事、飲水、歯磨きを済ませ、1時間前からは食事・飲水・歯磨き・ガム・喫煙など、口腔内に何かしらを入れることなく研究に参加していだだく。
3. 全体での研究説明・同意書記入の後、参加者背景とアンケートを記入していただき、唾液検査キットを配布する(前後チューブ2本)。この際、PSS-14日本語版にて参加者のストレス状態を把握する。また参加者に研究用のApple Watchを各自装着してもらい、あらかじめ各Apple Watchと1対1でペアリングした研究用iPhoneを起動し、研究用の生体データ取得アプリを起動し、生体データの取得を開始する。
4. その後封筒法にてランダムにヨガ瞑想・呼吸法介入群10名、対照群10名に分かれる。この際、感性アナライザを用いた脳波計測を行う6名(介入群3名、対照群3名)についても同時に封筒法で選定する。
   (感性アナライザは、使用できる機器のレンタル個数が限られており、6名での実施とする)
5. 1回目(前値)の唾液を採取し、検体は氷冷保存する。
6. 介入群は角間の里１階多目的ホール、対照群は2階に移動する。
7. 介入群はヨガインストラクター/研究協力員の桑原りさ氏より20分間のヨガセッション(瞑想・呼吸法)を開始する。また同時刻において対照群は20分間、室内で主に座位で自由に過ごしてもらう。
8. 20分間のセッション終了後は、1階研修室に戻る
9. 2回目(後値)の唾液を採取し、検体は氷冷保存する。(主要評価項目)
10. Apple WatchとペアリングしたiPhoneの生体データ取得アプリを停止し、Apple WatchとiPhoneを回収する。
11. 謝礼のAmazonギフトカードを配布して、介入群10名は解散とする。
12. 対照群参加者のうち希望者については、引き続いて桑原りさ氏よりヨガ瞑想・呼吸法の20分間のヨガセッションを追加して受けることを可能とする。この希望参加者が受けるヨガセッションの前後においても、前述の研究の方法の5.-9.に感性アナライザでのデータ取得も行い、ヨガセッション前後の検査・生体データの評価を行う。
13. 対照群希望者のヨガセッションと検体採取が終了して、全体解散。

　検査結果の開示について、本研究は主に健常成人を対象としたヨガ瞑想におけるストレスの変化を客観的に測定・観察する研究であり、得られた結果の精度は十分ではなく、結果を開示することで研究対象者及び血縁者に有益になることは少なく、かえって誤解や不安を招く懸念がある。このため，現時点では個別の結果は開示しない。

# **７．観察・検査・報告項目**

**（１）観察・取得項目**

| **基本情報** | 年齢、性別、職業、社会的因子(独居、同居)  併存疾患の有無(高血圧症、糖尿病、脂質異常症)、飲酒の有無と程度、喫煙の有無と程度 |
| --- | --- |
| **アンケート** | 主観的ストレススコア(Perceived Stress Scale (PSS-14) 日本語版を使用) |
| **唾液検査** | コルチゾール、α-アミラーゼ |
| **スマートウォッチ** | Apple Watch計測情報(脈拍数、心拍変動) |
| **感性アナライザ** | 脳波から推定された0-100スケールの  “興味”、”好き”、”ストレス”、”集中”、”沈静“の各項目 |

**（２）検査スケジュール**

1. ベースライン(前値)検査項目

　・基本情報：年齢、性別、職業、
　 社会的因子(独居、同居)、併存疾患の有無(高血圧症、糖尿病、脂質異常症)、
　　　　　　 飲酒の有無と程度、喫煙の有無と程度

　・アンケート：Perceived Stress Scale (PSS-14) 日本語版

　・唾液検査：コルチゾール、α-アミラーゼ

2. 介入(ヨガセッション/座位)中の検査項目

　・スマートウォッチ：脈拍数、心拍変動

　・感性アナライザ：“興味”、”好き”、”ストレス”、”集中”、”沈静“の各項目

3. 介入後(後値)検査項目

　(対照群のヨガセッション追加希望者のセッション後の検査項目もこれに準ずる)

　・唾液検査：コルチゾール、α-アミラーゼ

# **８．有害事象の評価と報告**

（１）有害事象の定義と報告方法

☒ 該当なし

（２）研究対象者に生じる負担並びに予測されるリスク及び利益、これらの総合的評価並びに当該

負担及びリスクを最小化する対策

　①予測される利益

　　本研究に参加することにより、研究対象者個人へは研究参加の謝礼としてAmazonギフトカード

　　1000円分を支給する。研究の成果はメンタルヘルス分野の研究開発とストレス応答の生理学的

　　機序解明に有益となる可能性がある。

　②予測されるリスクと不利益

　　本研究で取得する検査項目は、問診・アンケート調査、唾液検査、ウェアラブルデバイス等より非侵
　襲的に取得する生体データであり、介入についても通常サービスとして行われているヨガにおける呼吸
　法であるため、研究参加に伴う不利益はない。ただヨガの前後においては、研究対象者の体調をよ
　く確認し，不調があれば研究を中止する。

# **９．評価項目(アウトカム)**

**1. 主要評価項目**

　ヨガセッション終了後の唾液中コルチゾール値のベースラインからの変化量

**2. 副次評価項目**

　・ヨガセッション終了後の下記項目のベースラインからの変化量

　　1) 唾液中α-アミラーゼ
　　2) 脈拍数
　　3) 心拍変動
　　4) 感性アナライザ取得項目(“興味”、”好き”、”ストレス”、”集中”、”沈静“)

# **１０．統計的事項**

　本研究の参加者背景ならびに主要評価項目・副次評価項目は、まず記述統計にてデータをまとめる。その後、主要評価項目である「介入後の唾液中コルチゾールのベースラインからの変化量」については、両群の変化量について*t*検定を用いて群間比較を行う。また、コルチゾールの日内変動を考慮し、唾液採取時間を共変量とした共分散分析(ANCOVA)も検討する。副次評価項目においては、それぞれに適切な統計学的解析手法を用いて記述統計ならびに群間比較を行う。具体的には連続変数は*t*検定あるいは*Mann-Whitney U*検定、カテゴリー変数はχ二乗検定あるいはフィッシャーの正確検定、また回帰分析を含む多変量解析その他適切な共変量を加えた解析(ANCOVA等)と検討も行う。

# **１１．症例報告書の記入と報告**

　症例報告書は作成しない。

# **１２．倫理的配慮**

（１）遵守する倫理指針や法令

　本研究に携わるすべての者は、人を対象とする全ての医学研究が準拠すべき「世界医師会ヘルシンキ宣言」及び「人を対象とする生命科学・医学系研究に関する倫理指針」（文部科学省・厚生労働省・経済産業省）の内容を熟読し理解した上で遵守し、研究を施行する。

（２）個人情報の保護の方法

方法：研究対象者のデータや検体から氏名等の特定の個人を識別することができることとなる記述等を削り、代わりに研究用仮IDをつけたデータを保存・解析する。なお研究対象者とこの符号（番号）を結びつける対応表は金沢大学の個人情報管理者(野村章洋)が別途保存する。

　研究に関わる関係者は，研究対象者の個人情報保護について、適用される法令、条例を遵守する。また関係者は，研究対象者の個人情報およびプライバシー保護に最大限の努力を払い、本研究を行う上で知り得た個人情報を正当な理由なく漏らさない。これは関係者がその職を退いた後も同様とする。

個人情報は、個人情報管理者のみがパスワードを知る電子媒体に保管する。

　学会や学術誌で成果を報告する際には個人が特定されないように十分に配慮する。研究の中止又は終了後、学会発表、論文発表のうち、最も遅い時期から、研究に関する電子データ及び実験・観察ノートは10年、その他研究データ等は5年保存する。

# **１３．インフォームド・コンセントを受けるための手続きについて**

　研究についての説明を行い、十分に考える時間を与え、参加者が研究の内容をよく理解したことを確認した上で、研究の参加について依頼する。参加者本人が研究参加に同意した場合、同意文書を用い、参加者本人による署名を得る。研究担当者は同意文書に、説明を行った医師名と説明日、説明を受け同意した参加者名、同意日の記載があることを確認する。

　同意文書は2部コピーし、1部は参加者本人に手渡し、１部は研究責任医師が電子カルテ等に保管する。原本は実施機関で定められた保管場所に保管する。

　なお、本研究に本学の学生，教職員が研究の参加を拒否・撤回した場合でも，今後の学生生活に影響することはない。

# **１４．研究対象者に生じる費用負担について**

　本研究の参加者にはAmazonギフトカード1000円分を謝礼として支給する。本研究の参加に伴い交通費が発生する場合，研究参加者の自己負担とする。

# **１５．本研究に係る資金ならびに利益相反について**

　本研究は、金沢大学先端観光科学研究所の助成金を得て実施する。本研究の計画・実施・報告において、研究の結果および解釈に影響を及ぼすような「起こりえる利益相反」は存在しないこと、および研究の実施が研究対象者の権利・利益を損ねることがないことを確認する。

また本研究の研究担当者は、この研究において企業との間に利害関係はない。本研究の研究担当者は、金沢大学の規定に基づく利益相反審査機関へ自己申告し、その審査と承認を得るものとする。

# **１６．実施計画の変更について**

　研究の進捗にともない、研究内容及び研究組織・期間などに計画の変更の必要が生じた場合は、金沢大学医学倫理審査委員会の承認を得て、変更を行う。

# **１７．試料・情報について**

（１）試料・情報の種類、保存、記録、破棄について

A．人体から取得した試料

☒該当あり

試料の種類：唾液検体

保存・破棄について：

　研究責任者は，定められた保管方法に従って研究分担者等が適切に保管するよう指導し，試料の

　漏えい，混交，盗難，紛失等が起こらないよう必要な管理を行う。採取した唾液は，研究終了後

　直ちに破棄する。廃棄の際は個人情報に注意して廃棄を行う。

試料及び情報の二次利用について：

　本研究で得られた試料そのものの二次利用は行わない。

B．情報

☒該当あり

情報の種類：問診・アンケートによって得られた参加者基礎情報、唾液検体解析結果、感性アナライザから得られた脳波解析情報

保存・破棄について：

　研究責任者は、定められた保管方法に従って研究分担者等が適切に保管するよう指導し、情報の

　漏えい、盗難、紛失等が起こらないよう必要な管理を行う。電子データ及び実験・観察ノートは研究終

　了もしくは中断または，論文等が発表されてから遅い時期から10年間、その他の研究データ等は5

　年間保存した後、破棄する。

試料及び情報の二次利用について：

本研究で研究対象者から取得した情報については、同意を受ける時点では特定されない将来の研究のために用いる可能性がある。その場合には，新たな研究計画について金沢大学倫理審査委員会の審査を受けたうえで，別途研究対象者に説明した上で実施する。

保存の責任者について：情報は研究代表者 野村章洋が保管する。

（２）試料・情報の他機関との授受の記録について

【他機関に試料・情報を提供する場合（業務の一部委託による提供を含む）】

☒該当あり

①提供記録の作成方法

　本研究計画書を提供記録とし，変更時は変更申請で対応する。

②提供記録の保管方法・提供記録の保管場所：金沢大学附属病院・電子申請システム

③提供先の機関名称：フナコシ株式会社

④提供先の責任者名：代表取締役社長 池田哲也

⑤提供する試料・情報の項目：唾液検体

【他機関から試料・情報の提供を受ける場合】

　該当なし

# **１８．部局長への報告**

　☒　有害事象報告（随時）

　☒　研究計画書からの重大な逸脱に関する報告（随時）

　☒　実施状況報告（年１回）

　☒　終了報告（研究終了時）

# **１９．研究成果の帰属と結果の公表**

　本研究成果ならびに知財等が得られた場合、その権利は金沢大学ならびに本研究従事者に帰属し、研究参加者にはこの知的財産権は属さない。研究成果は国内外の学会及び学術誌に公表する場合がある。

# **２０．研究実施体制**

**金沢大学(附属病院)における研究実施体制**

　研究責任者　融合研究域融合科学系 准教授 / 附属病院循環器内科 野村 章洋

　研究分担者　融合研究域融合科学系 教授 / 先端観光科学研究所 所長 堤 敦朗

融合研究域融合科学系 助教　 森崎 裕磨

先端観光科学研究所 研究員 周 英

先端観光科学研究所 研究員 陳 萍

　研究協力者 全米ヨガ協会認定RYT200ヨガインストラクター 桑原 りさ

先端観光科学研究所 特任准教授・コーディネーター 今 洋佑

# **２１．研究業務の一部を委託する場合の当該業務内容及び委託先の監督方法**

（１）唾液検査の外部委託

　会社名：フナコシ株式会社

　会社代表者氏名：代表取締役社長 池田哲也

　会社担当者氏名：野津裕佑

　住所：〒113-0033　東京都文京区本郷2-9-7

　電話番号：03-5684-1645

　業務内容：唾液中のコルチゾール、α-アミラーゼの測定委託

　金沢大学による業務先への監督方法：検査委託契約において、金沢大学が定める個人情報保護

　等の安全管理措置の内容に照らし、遵守されなかった場合の対応等を確認して委託する。

# **２２．モニタリングについて**

　該当なし

# **２３．監査について**

　該当なし

# **２４．相談窓口**

　研究対象者等及びその関係者からの相談等への対応窓口として、研究事務局が対応する。

住所　〒920-1192　石川県金沢市角間町

担当　融合研究域融合科学系 / 先端観光科学研究所　野村章洋
E-mail: anomura@med.kanazawa-u.ac.jp

# **２５．文献**

1. 光武 幸. ウェルネスツーリズム-健康と美を求めて現代的観光-. 創風社 2010.

2. 国立大学法人琉球大学 国際地域創造学部 ウェルネス研究分野. ウェルネスツーリズムとは. URL from: https://health-tourism.skr.u-ryukyu.ac.jp/wellness-tourism/.

3. Carney DR, et al. Power Posing: Brief Nonverbal Displays Affect Neuroendocrine Levels and Risk Tolerance. Psychol Sci. 2010 Oct;21(10):1363-8. doi: 10.1177/0956797610383437. Epub 2010 Sep 20. PMID: 20855902.

4. Hunter MR, et al. Urban Nature Experiences Reduce Stress in the Context of Daily Life Based on Salivary Biomarkers. Front Psychol. 2019 Apr 4;10:722. doi: 10.3389/fpsyg.2019.00722. PMID: 31019479; PMCID: PMC6458297.
